# Rare Tumor-Normal Matched Whole Exome Sequencing Identifies Novel Genomic Pathogenic Germline and Somatic Aberrations

**DOI:** 10.3390/cancers12061618

**Published:** 2020-06-18

**Authors:** Ryan Sprissler, Bryce Perkins, Laurel Johnstone, Hani M. Babiker, Pavani Chalasani, Branden Lau, Michael Hammer, Daruka Mahadevan

**Affiliations:** 1 Department of Health Sciences, Center for Applied Genetics and Genomic Medicine, University of Arizona, Tucson, AZ 85721, USA; ryans1@arizona.edu; 2 Arizona Research Labs, University of Arizona Genetics Core, University of Arizona, Tucson, AZ 85721, USA; laureljo@arizona.edu (L.J.); bmlau@email.arizona.edu (B.L.); 3 Department of Medicine, Division of Hematology and Oncology, University of Arizona Cancer Center, University of Arizona, Tucson, AZ 85724, USA; bperkins@deptofmed.arizona.edu (B.P.); hanibabiker@email.arizona.edu (H.M.B.); pavanic@email.arizona.edu (P.C.); 4 Department of Medicine—Hematology/Oncology, University of Texas Health San Antonio, Mays Cancer Center, San Antonio, TX 78229, USA

**Keywords:** rare tumors, whole exome sequencing, tumor-germline matched sequencing, inherited variants, copy number alteration (CNA), double hits

## Abstract

Whole exome sequencing (WES) of matched tumor-normal pairs in rare tumors has the potential to identify genome-wide mutations and copy number alterations (CNAs). We evaluated 27 rare cancer patients with tumor-normal matching by WES and tumor-only next generation sequencing (NGS) as a comparator. Our goal was to: 1) identify known and novel variants and CNAs in rare cancers with comparison to common cancers; 2) examine differences between germline and somatic variants and how that functionally impacts rare tumors; 3) detect and characterize alleles in biologically relevant genes-pathways that may be of clinical importance but not represented in classical cancer genes. We identified 3343 germline single nucleotide variants (SNVs) and small indel variants—1670 in oncogenes and 1673 in tumor suppressor genes—generating an average of 124 germline variants/case. The number of somatic SNVs and small indels detected in all cases was 523:306 in oncogenes and 217 in tumor suppressor genes. Of the germline variants, six were identified to be pathogenic or likely pathogenic. In the 27 analyzed rare cancer cases, CNAs are variable depending on tumor type, germline pathogenic variants are more common. Cell fate pathway mutations (e.g., Hippo, Notch, Wnt) dominate pathogenesis and double hit (mutation + CNV) represent ~18% cases.

## 1. Introduction

Genomic analysis of tumors has dramatically reshaped cancer treatment through the identification of genetic variants that provide diagnostic and prognostic information and that aid in therapeutic selection [1]. Patients with rare cancers generally present with advanced disease and for most there is no standard of care therapy. This results in poor 5-year survival rates compared with the more common cancers [2]. While rare tumors are individually infrequent, combined together they comprise nearly 25% of all cancer cases [3]. In the U.S. rare tumors are defined as those that are less than 15 per 100,000 cases. Here we follow however the stricter guidelines of the European tumor classification group (RARECARE) of less than 6 per 100,000 per year [4]. The low frequency of these cases presents unique challenges in pathological classification, presentation and progression of disease and by extension, therapeutic options for clinical management.

Research to identify causes or to develop strategies for prevention or early detection are also difficult. Despite these challenges, rare tumors offer the opportunity to identify novel targets and signaling pathways [5]. A recent precision medicine study focused on molecular characterization of rare cancers identified actionable variants over 92% of the time, with 52% receiving a matched therapy [3]. Most patients receiving these therapies achieved stable disease, with a smaller number achieving partial or complete remission. Additionally, a high percentage of rare tumors are known to harbor pathogenic germline variants, for example, 29% of pheochromocytomas, 14.1% of pancreatic ductal adenocarcinomas, and 12.5% of soft tissue sarcomas [6,7,8,9,10]. However, the majority of cancer cases undergoing genetic analysis are sequenced via tumor-only gene panels (e.g., Caris Life Sciences, Foundation Medicine) that fail to identify genes outside of their search space, generate poor detection of copy number alterations (CNAs), and are often unable to distinguish if a variant is in the germline or of somatic origin [11].

In this study, we performed tumor and matched germline whole exome sequencing (WES) of rare tumors from 27 patients that were evaluated at our Early Phase Therapeutics Program. Each patient also had a biopsy sample tested using a clinically available tumor-only assay from a commercial vendor for comparison. Our aim was to further characterize and compare both differences and commonalities of rare and common cancers, to better inform prognosis, selection of potential therapeutic pathways and design trials for rare cancer patients.

## 2. Results

### 2.1. Somatic and Pathogenic Germline Variants Detected in the Rare Tumor Cohort

**Figure 1:**
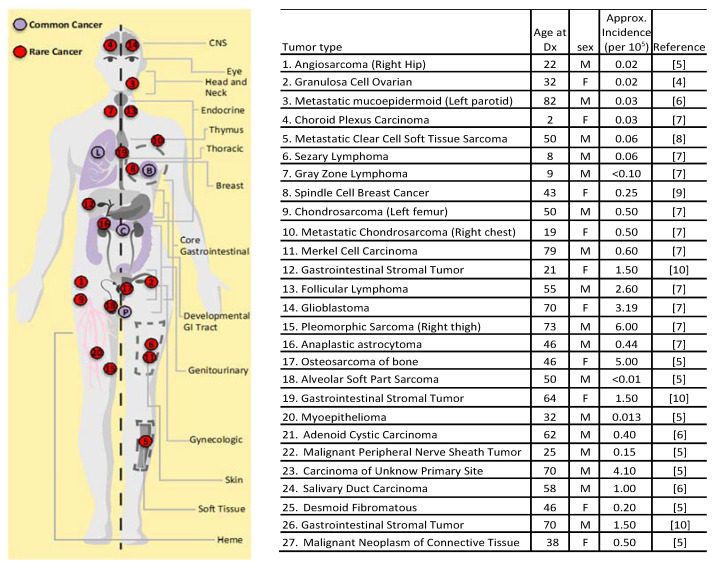
Approximate locations of rare tumors. Rare tumor types as numbered in Table 1 are shown in red dots with age at diagnosis, gender and incidence. Tumors of the blood or lymph are shown in one location labeled ‘heme’, while tumors of skin are shown in dashed box labeled ‘skin’ and tumors of soft tissue are shown in dashed filled box labeled ‘soft tissue’ (on left leg). Common cancer types used for comparisons are shown in lavender: Lung (L), Breast (B), Colon (C), and Prostate (P). Gray zone lymphoma (#7)—lymph node; GIST (#12)—Stomach; 13 Follicular lymphoma (#13)—lymph node; Primary mediastinal lymphoma (#19)—between the 2 lungs in the mediastinum [4,12,13,14,15,16,17].

Twenty-seven rare cancer cases (mean age: 46.0 years, range: 2–82 years; 17 males, 10 females) were evaluated with whole exome sequencing (WES) for tumor and germline. There were 23 different rare cancer types represented in our survey, all of which met the criteria of a rare cancer (Figure 1).

Using the WES data, we detected a total of 3343 germline single nucleotide variants (SNVs) and small indel variants. Of these, 1670 were found in oncogenes and 1673 were in tumor suppressor genes, generating an average of 124 germline variants per case (Table S1). This likely includes many benign polymorphisms as a maximum population frequency of 5% was used in the filtering criteria for germline variants (see Methods). The number of somatic SNVs and small indels detected in all cases totaled 523, with 306 in oncogenes and 217 in tumor suppressor genes (Table S2). Classification of oncogenic genes (OG) and tumor suppressor genes (TSG) was defined by the Oncogene Database (http://ongene.bioinfo-minzhao.org/) and the Tumor Suppressor Gene Database (https://bioinfo.uth.edu/TSGene/). All 694 oncogenes and 1016 tumor suppressor genes (129 genes were classified as both OGs and TSGs) are listed (Table S3). Variants were then further categorized as either a passenger mutation or a functional driver mutation using the Cancer Genome Interpreter tool (https://www.cancergenomeinterpreter.org/home). Of the variants deemed to be driver mutations, there were a total of 72 germline variants (2.67/case, 48 in TSGs/24 in OGs) and 22 somatic variants (0.81/case, 14 in TSGs/8 in OGs) (Table 1). From germline variants found to be in cancer predisposition genes, six (all heterozygous) were identified to be pathogenic or likely pathogenic by the more stringent ACMG guidelines: *BRCA2*-Q2859Kfs (gray zone lymphoma), *SDHA*-R75* (spindle cell breast cancer), *SDHC*-A3Rfs (gastrointestinal stromal tumor), *RUNX1*-M151L (glioblastoma), *FANCC*-c.456+4A>T splice site/exon skipping (anaplastic astrocytoma), and *MUTYH*-G396D (alveolar soft part sarcoma) (Table 2).

Among the 27 rare tumor patient cases included in our study, there were a total of 27 actionable somatic variants. Eight of the 27 patients in which we identified a variant of clinical significance had multiple actionable variants. One of the three tumors without an actionable variant—Merkel Cell Carcinoma (MCC), stained positive for Merkel cell polyoma virus and therefore was expected to have a very low tumor mutation burden [18]. Interestingly the *SPEN*-Q3621* nonsense variant that was identified in this tumor likely truncates the protein product (a hormone inducible transcriptional repressor), resulting in loss of a portion of a domain that is necessary for interactions with other nuclear co-repressors. While *SPEN* mutations are reported in 8% (8/97) of MCC samples in COSMIC, this variant is not functionally characterized and its effect on protein function is unknown.

Actionable variants found in the patient cohort were classified in the four categories: FDA-approved for rare cancer type, FDA-approved for different cancer type, clinical trial for rare cancer type, clinical trial for different cancer type. All reported variants were deemed to be somatic.

**Figure 2:**
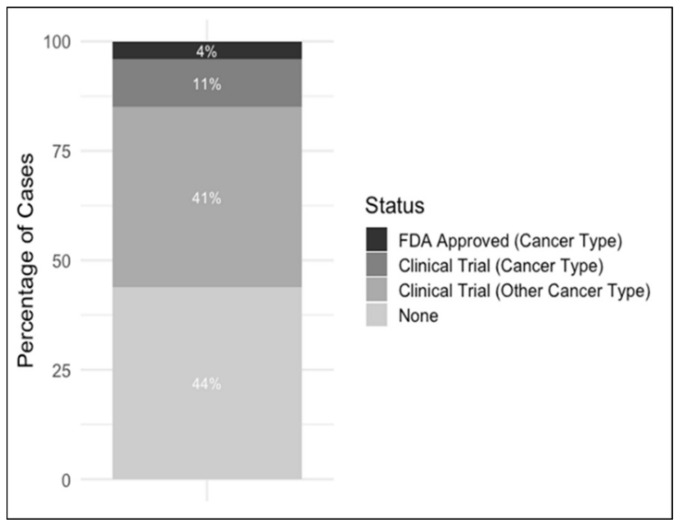
Percentage of cases with actionable variants.

**Figure 3:**
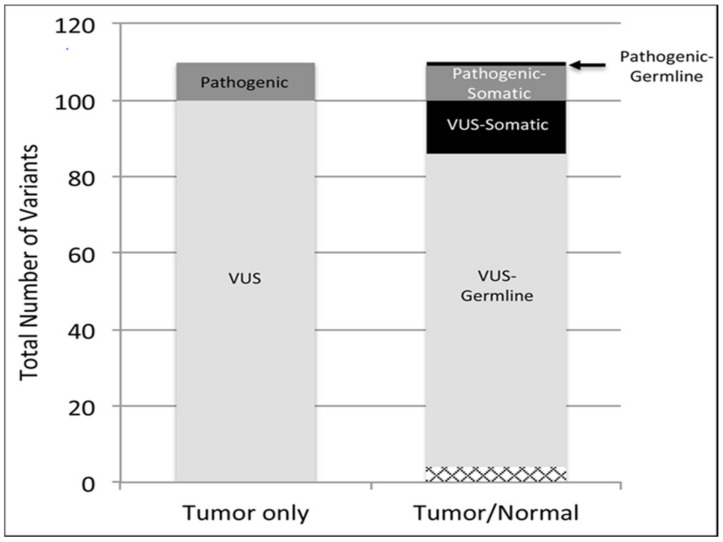
Variants classified as pathogenic or variant of uncertain significance (VUS) in tumor only versus tumor-germline sequence analysis in the seven cases for which both datasets were available (case # 1, 2, 3, 5, 11, 12, and 15 in Table 1). In the latter case, variants could be further classified as somatic or germline in origin. Cases of missing variants are shown at the bottom (hatched box).

Unsurprisingly, for the majority of rare tumor patients in our study, available FDA approved medications and/or clinical trials were not available for use in the patient’s tumor type. In fact, only one patient had a variant with an FDA approved therapy, and three had a variant with a clinical trial for their tumor type (Figure 2). This is illustrative of the vastly different treatments available for rare vs. common tumor types. Interestingly, 18 patients were able to gain access to targeted therapies, though this was often in an off-label capacity. When comparing our WES tumor/germline with tumor only analysis, there was general concordance in the classification of known pathogenic variants and VUS. However, while most of the pathogenic/likely pathogenic variants where found to be somatic using both assays, the vast majority (~80%) of the clinically reported VUS turn out to be inherited when using the tumor/germline analysis (Figure 3). This was evident in our patients who also underwent tumor only NGS with a 592-gene panel assay (Caris Life Sciences, Phoenix, AZ, USA).

Additionally, our tumor/germline approach identified a single pathogenic variant that turned out to be germline in origin. This frameshift variant (A3fs) in the C subunit of the succinate dehydrogenase (SDH) gene was found in a patient with gastrointestinal stromal tumor (GIST). This variant occurs early in the gene and is predicted to lead to nonsense mediated decay (loss of function), which generates oncometabolites that dysregulate epigenetic repression [19]. The patient was treated with olaparib (off-label) and after 2 months of therapy showed a 13.8% reduction in tumor size. As of the writing of this manuscript, the patient continues to take olaparib and has maintained stable disease for the past 7 months. Indeed, loss or markedly reduced SDHB expression has associated with familial cancer predisposition syndromes with affected individuals at increased risk for developing paragangliomas, pheochromocytomas (rare forms of adrenal tumors) and GISTs [20].

### 2.2. Germline Pathogenic Variants Are More Common in Rare Cancers

The percentage of cases harboring an inherited pathogenic variant appears to differ between rare vs. common (i.e., lung, breast, colon, rectal, and prostate) cancers (Table 3). Among our rare cancer cases, 22% had a pathogenic or likely pathogenic germline variant, consistent with previously published cases [14]. In contrast, common cancers taken from 3451 combined cases in TCGA, showed 7.9% of variants as germline pathogenic or likely pathogenic [14,21]. This increased rate of inherited pathogenic variants in rare cancer was found to be statistically significant using the Fisher exact test (*p* = 0.01800). Given the small sample size of our rare tumor cohort, we analyzed other rare tumors in these same databases to confirm the higher frequency of germline variants. There is a consistent yet slight increase in the overall percentage of germline variants detected in rare tumors in our cohort when compared to the other rare tumor cohorts (Table 4). Of note is the large variance in total percentage of pathogenic/likely pathogenic called germline variants stemming from a lack of consistency in methodology regarding pathogenic/likely pathogenic filtering criteria. This absence of a classification standard continues to be a confounding problem, creating issues in cross-study comparisons as well as general clinical variant reporting.

### 2.3. Copy Number Alterations Are Variable and Depend on the Rare Tumor Type

**Figure 4:**
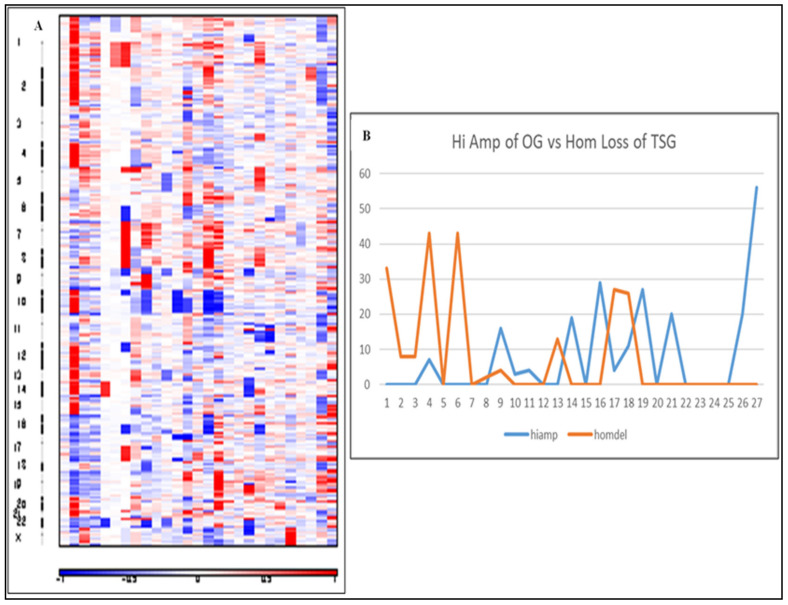
Copy number alteration (CNA) analysis of the 27 rare-cancer cases using GISTIC2 with input from Sequenza. (**A**). Heat map showing amplified (red) and deleted (blue) regions along the chromosomes. Cases are aligned on the *x*-axis and chromosomes on the *y*-axis. (**B**). Frequency of highly amplified oncogenes (blue) and tumor suppressor genes with putative homozygous deletions (orange) in each case.

Copy number analysis (CNA) performed using Sequenza [22] on all WES cases and analyzed using the GISTIC2 pipeline [23], produced a heatmap of the overall amplification and deletion scores across all chromosomes to compare cases (Figure 4A). These results were later compared to TCGA GISTIC gene-level CNA files downloaded from the cBioPortal [24]. GISTIC distinguishes two levels of amplification: a low-level gain (“amp”) and a high-level amp (“hiamp”) that is often a focal copy number increase; likewise, it outputs two levels of deletion, a shallow loss (“del”) that may be a heterozygous deletion and a deep deletion (“homdel”) that is likely to be a homozygous loss of the gene. As shown the total number of amplified oncogenes and deletions of tumor suppressor genes in our cohort was widely variable from case to case (Table S4). Nine of the 27 cases contained at least one amplification of an oncogenic gene or loss of a TSG. Of the cases containing amplification of oncogenic genes (~50% cases), the average was eight oncogenes/case (min 3, max 56). When comparing to loss of TSG, only about a third of cases showed a loss with an average of 20.7 tumor suppressor losses/case (min 2, max 43). Interestingly, there appears to be a negative correlation between the presence of a high-level amplification in an OG vs. a deep deletion of a TSG (Figure 4B). There was negative association between having an increase in the number of “high-amp” of OG with an increase in the number of “homdel” TSGs.

**Figure 5:**
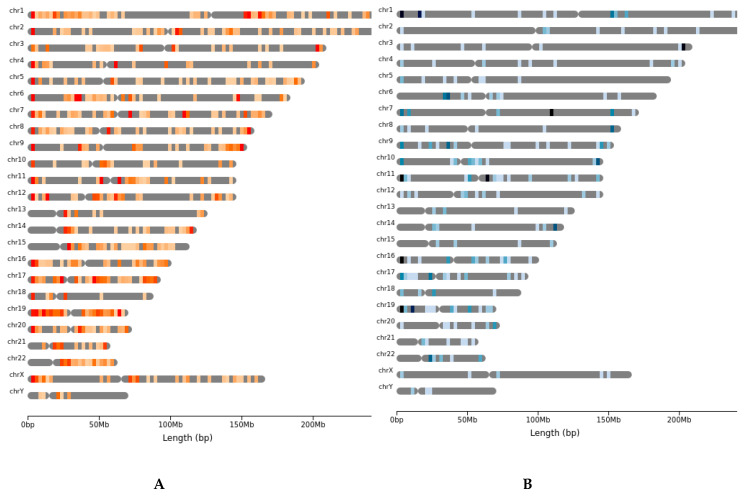
CNA from Sequenza, plotted using the chromoMap package, for the 27 rare cancer cases. (**A**) Heat map showing frequency of amplified regions along the chromosomes. (**B**) Heat map of deleted regions along the chromosomes.

When mapping the locations of all CNVs on to each chromosome, we find that there appears to be several protected regions as well as hot spots incurring gains and losses of copies at a much greater frequency. While all chromosomes showed some level of amplification, there is a greater frequency of amplification events clustering near the tips of both the p and q arms (Figure 5A). There was a similar clustering of loss of copy at the tips of each chromosome, albeit at a lower total count (Figure 5B). While there was a higher total count of amplifications overall, there were several consistencies between the mapped gains and losses. No gains or losses were detected on the short arms (p) of chromosomes 13, 14, 15, 22, and in both cases the full chromosome 19 appears to undergo the most amplifications and losses. Many of the usual hot spots seen in other analysis also appear to be affected in rare tumors [25,26,27].

We used the TCGA containing 3149 common and 2120 rare cancers showed amplifications are more prevalent than deletions: rare (2.25-fold) and common cancers (3-fold) (Figure S1A). Common cancers have an overall greater number of amplifications (2.8-fold) and deletions (2.2-fold) than rare cancers. When this analysis is limited only to per sample amplifications of oncogenes and deletions of tumor suppressor genes, the same trend is seen (Figure S1B,C). Whether these differences could be attributed to a specific cancer type, we graphed the total number of amplifications and deletions per sample for all protein coding genes across all of the rare and common cancer types individually (Figure S2A).

### 2.4. Cell Fate Pathways and TP53 Mutations Dominate Pathogenesis in Rare Cancers

**Figure 6:**
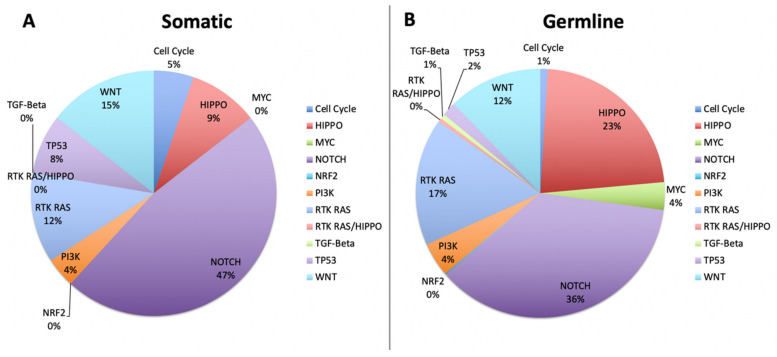
The percentages of both somatic and germline variants detected in the rare tumor cohort. The largest difference between somatic and germline variants is seen in the increase of germline variants in the Hippo pathway (9% somatic vs. 23% germline).

To determine the alteration frequencies of pathways known to be involved in cancer, we mapped all variants to genes in 11 canonical signaling pathways [28]. Figure 6 shows the percentages of both somatic and germline variants detected in our rare tumor cohort. The largest difference between somatic and germline variants is seen in the increase of germline variants in the Hippo pathway (9% somatic vs. 23% germline), involved in cell proliferation and differentiation. The Hippo pathway consists of 12 tumor suppressor genes and three oncogenic genes that determines cell fate (CF) and is known to be disrupted at low frequencies in several common cancers, most notably CNS and gastrointestinal tumors [28]. Outside of the increase in Hippo signaling, disruption of the germline variants largely matched the somatic variants with both groups showing Notch signaling, another CF determinant, as the most frequently hit with 36% and 47% respectively. Interestingly, all 27 cases had germline variants detected in the RTK-RAS pathway key to cell survival (CS) (101 total variants), Hippo (138) and Notch pathways (238), and 24 of 27 cases showed a variant in the WNT pathway (75 total) which, excluding the RTK-RAS pathway, are all largely comprised of TSGs. When looking at somatic variants, 20 cases had at least one variant in the Notch pathway (36 total), while eight cases had a variant in the WNT pathway (11 total), both CF determinants.

**Figure 7:**
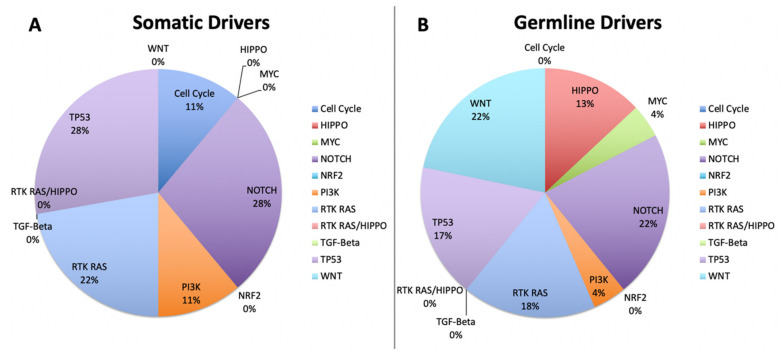
Analysis limited to only variants predicted to be driver mutations, 22% of germline variants are in the WNT signaling pathway and 13% in the Hippo pathway, with no somatic driver variants detected.

When the analysis was limited to only variants predicted to be driver mutations, we saw 22% of germline variants in the WNT signaling pathway and 13% in the Hippo pathway, with no somatic driver variants detected (Figure 7). All other pathways in the driver mutation analysis were relatively consistent across germline and somatic except for *TP53*, which saw an increase of 17% to 28% from germline to driver. The number total of cases showing a predicted germline driver mutation was limited to 23, with (*n* = 5) cases each showing variants in WNT, Notch (*n* = 4) cases and each in RTK-RAS and *TP53* (*n* = 3) in Hippo. No other pathway showed a germline driver mutation in more than 1 case. Of the somatic variants, the predicted drivers clustered around the Notch and p53 pathways with four and five respectively, with one case showing a double hit in *TP53*. We also saw three cases with a somatic driver in the RTK-RAS pathway, one of which also had a double hit.

### 2.5. Double Hit Analysis Shows Driver Oncogenes Correlate to CNAs

Finally, we investigated if any variants classified as drivers had also undergone any additional copy number variation. This analysis identified 5 total cases (18.5%) experiencing a double hit (Table S5). Of the eight total driver mutations found in oncogenes, four underwent amplification. The most severe of these likely to have a functional effect was found in the anaplastic astrocytoma case, which saw both a driver mutation (rs149840192, p.A289V) and an amplification of at least five extra copies of the EGFR. This was of particular interest as both amplifications and mutations in EGFR have been shown to be drivers in many cancer types and may confer efficacy of treatment with tyrosine kinase inhibitors [29,30]. With respect to TSG double hits we identified only one case, metastatic chondrosarcoma, that contained both a driver mutation and a CNA. This was a stop gain variant that also showed a heterozygous loss of copy in *TP53*.

## 3. Discussion

Deep genomic profiling of rare tumors is critical to understand the molecular architecture of each specific cancer type to attain clear and actionable therapies due to difficulties in early diagnosis and limited or no standard of care therapies [31]. Tumor-only NGS fails to provide a complete picture since germline sequencing is absent which can generate false positive biomarkers, that may lead to targeting of a variant unrelated to cancer development [32] and/or not be present on the panel. Our findings that rare tumors have an increased rate of germline pathogenic variants compared to common tumors (Table 4) driven by genetic predisposition(s) as opposed to environmental or hormonal factors, increase the lifetime risk for developing cancer. This may help explain the wide tissue distribution affected by rare cancers (Figure 1). While demonstrated in a relatively small initial sample size, this finding warrants further investigation with tumor/germline sequencing of more rare tumor cases. Such studies may have implications for cancer risk assessment and potential genetic counseling of patients and family members in the setting of rare tumor diagnoses [33].

For example, in a patient with glioblastoma, tumor-normal match pair WES identified a germline variant *RUNX1*-M151L deemed likely pathogenic (ACMG guidelines [34]) but not reported with tumor-only NGS (Caris Life Sciences) as it was not included in their panel. *RUNX1* has been described as an activator of gene expression and a positive driver of the GBM mesenchymal aggressive phenotype [35]. Over-expression of *RUNX1* in U87 GBM cells inhibited tumor growth by extensive down-regulation of target genes and deregulation of key developmental pathways [36]. Given the aggressive phenotype of our patient, we can surmise that the *RUNX1*-M151L variant has a partial or complete loss of function. In addition, there are important implications for family members who wish to evaluate their risk, and given knowledge of germline predisposition may opt for enhanced cancer screening and prevention strategies [37,38].

Our cohort of rare tumors affected 18 different tissues of the body (Figure 1), with only one case affecting the tissues typical of common cancers (one case of spindle cell breast cancer—chosen for comparison (i.e., lung, colon, breast or prostate)). We identified *IDH1* and *PIK3CA* gene variants which occurred at the same site. The *IDH1* variant occurred at amino acid 132 in the *IDH1* gene in two sarcoma cases; chondrosarcoma R132C and pleiomorphic sarcoma R132G substitution respectively. The pleomorphic sarcoma with a concurrent *IDH1* R132G mutation and a *SUFU* splice site mutation may indicate that the Hedgehog (Hh) pathway is operant in these cells independent of SHH ligand expression [39] since SUFU is a negative regulator of Hh signaling [40]. Further, a frameshift mutation in *SUFU* was found in our metastatic mucoepidermoid sarcoma patient indicative of active Hh signaling amenable for therapeutic intervention. The mutant *IDH1* makes it a highly promising candidate for the IDH1 small molecule inhibitor ivosidenib [41] approved for AML. The *PIK3CA* variant occurred at amino acid E542K in two rare salivary gland tumors, myoepithelioma and mucoepidermoid tumor. A concurrent *HRAS* mutation identified in salivary mucoepidermoid tumor patient will most likely not respond to a PI3KCA inhibitor, however, the myoepithelioma patient could benefit from off-label therapy. There were nine genes in our cases that occurred in more than a single rare cancer case of which *TP53* is the most prevalent occurring in seven cases. *TP53* is the most common gene to carry pathogenic variants in common cancers with an average of 44.4%. In addition, we found two relatively common cancer genes in more than one rare cancer case: *ARID1A* (occurred in two cases and twice in one case) and *CDKN2A* (occurred in two cases). *ARID1A* and *CDKN2A* are also on the top 20 cancer gene list in breast, colon, and lung cancers, and are found in an average of 7.4% and 4.2% cases, respectively, across all four common cancer types. Both *ARID1A* and *CDKN2A* mutations are context dependent tumor suppressor genes that may be targetable in a synthetic lethal pair such as with a EGLN (prolyl hydroxylase) inhibitor (targeting HIF1α) or *MTAP* deletions with a PRMT5 (arginine N-methyltransferase 5) inhibitor dependent tumors respectively [42]. To look for additional commonality across cases we performed a GO enrichment analysis which showed 32 significant GO term associations (Table S6).

Further, we found six genes—*FOXL2, TNFAIP3*, *CHD1L, HRAS, KIT, CTCF*—that occurred in 12 cases that are *not* on the top 20 list of any of the four common cancers used for comparison. These genes are found in <5% of breast, colon, prostate, or lung cancer cases. *FOXL2* mutations was found in Granulosa cell ovarian cancer case, ~5% of ovarian cancer, which functions as a DNA binding forkhead transcription factor required for granulosa cell differentiation. The Cys134Trp mutation in *FOXL2* is associated increased cell cycling and downregulation of genes associated with apoptosis [43]. The tumor suppressor-oncogene pair *TNFAIP3* [44] and *CHD1L* [45] both have a frameshift deletion respectively is found in our gray zone lymphoma patient. Both play pro-oncogenic roles in cancer: *TNFAIP3* is a ubiquitin-editing enzyme that binds and inhibits E3 ubiquitin ligase RNF168 responsible for regulating histone H2A turnover key to proper DNA repair, while *CHD1L* is a DNA helicase possessing chromatin remodeling functions important for early embryonic development and promotes cell proliferation and anti-apoptosis. The GIST patient also has a tumor suppressor-oncogene pair, *CTCF* [46] and *KIT* [47] both with a non-frameshift deletion respectively. CTCF is a 11-ZF DNA binding protein known as the master weaver of the genome as it functions in the regulation of chromatin structure and function. It is a haplo-insufficient tumor suppressor gene with of loss of function leading loss of epigenetic silencing and anti-apoptosis [46]. Oncogenic *KIT* mutations (e.g., non-frameshift deletions) are well established in GIST. We are the first to document a concurrent loss of *CTCF* and a *KIT* activating mutation in GIST, however, in *SDH*-deficient GIST, CTCF may be epigenetically silenced [48].

Copy number alterations (CNAs) play a role in cancer type (e.g., breast, colorectal) [26], tumor progression, overall prognosis, and response to therapy [27]. Rare cancers individually have a generally low level of amplifications, on par with colorectal and prostate cancers, with the dramatic exception of sarcomas and uterine carcinomas (384/case and 541/case respectively), which show amplifications at an equal or higher level than lung and breast cancers (343/case and 385/case respectively) (Figure S1A). Deletion frequencies were generally lower in the rare tumor cohort with the exception of sarcomas, which showed a number of per-sample deletions (133/case) that was higher than any other rare tumor types and higher than all common cancers save prostate (180/case) (Figure S1B,C).

Analysis restricted to amplifications of oncogenes and loss of tumor suppressor genes showed the same patterns in both rare and common cancers (Figure S2A). Sarcomas and uterine carcinomas showed a significantly elevated frequency of amplifications of oncogenes when compared to all other rare cancer types (Figure S2B). Further, sarcomas showed an increased frequency of deletions of tumor suppressor genes (Figure S2C). This has significant implications as previous studies have shown a correlation between CNA burden and immunotherapy response rates, particularly in lung squamous cell carcinoma and certain breast cancer subtypes [49]. Moreover, there have been recent reports showing that immunotherapies are well tolerated in advanced sarcoma and many patients are seeing clinical benefit [50]. Taken together, the continued characterization of CNA burden in rare tumors offers potential for better informed clinical management as well as a general prognostic indicator.

This study is limited by the relatively small sample size and by methodology that only targets the protein-coding regions of the genome. This limitation did not allow us to assess many important questions concerning the overall stability of some genomic regions compared to others in rare cancer, any impacts of epigenetic regulation, etc. In addition, there are difficulties in comparing rare and common cancers from different data sets. Despite these limitations our study suggests that tumor-germline matched analysis may have particular advantages in the case of rare tumors.

## 4. Materials and Methods

### 4.1. Patients with Rare Cancers

This study involved a consecutive group of rare tumor patients referred to the Early Phase Therapeutics Program regarding molecular profiling-based basket and umbrella clinical trials and/or off-label therapies. All patients (*n* = 27, see Graphical Abstract) were evaluated at the University of Arizona Cancer Center Clinic and consented to an IRB approved protocol. All procedures performed, including buccal samples for germline DNA, involving human participants were done in accordance with the ethical standards of the University of Arizona Institutional Review Board (IRB#1505875499; Precision Medicine and Genomic Analysis Protocol for Oncology). Tumor biopsies for all patients were evaluated by University of Arizona Genetics Core (UAGC, Tucson, AZ, USA), where WES was performed. Paraffin blocks from the same tumor samples (without matched normal) were sent to Caris Life Sciences for NGS on a 592-gene panel (Caris MI/X platform).

### 4.2. Tumor and Germline Whole Exome and Gene Panel Sequencing

Tumor genomic DNA was isolated from paraffin tissue blocks using the ReliaPrep FFPE gDNA Miniprep system (Promega, Madison, WI, USA). Matching germline DNA was isolated from buccal swabs. DNA was quantified using the Qubit quantitation system with standard curve as per the supplier protocol (Thermo Fisher, Waltham, MA, USA) and all samples were further tested for quality using the Fragment Analyzer (Advanced Analytical, Ames, IA, USA) following the manufacturer-recommended protocols. The estimated fraction of tumor cells in the selected tissue ranged from 10% to 90%. Whole exome sequencing (WES) was performed by array capture of 60 Mb of exome target sequence using the SureSelectXT Human All Exon V6 enrichment (Agilent, Santa Clara, CA, USA) or a Nextera kit (Illumina, San Diego, CA, USA). All exome library builds were followed by paired-end sequencing (2 × 100 bp reads) on an Illumina HiSeq 2500 or NextSeq 550.

### 4.3. Sequence Analysis

The mean coverage over the exon target regions was 154-fold for the tumor sample and 99-fold for the normal sample. Sequences were aligned to the human genome using standard methods [51], followed by variant calling comparing the tumor/germline samples using MuTect2 (GATK v.3.7, [52]) and freebayes (v0.9.7, [53]). BAM/VCF files generated for this study have been submitted to the database of Genotypes and Phenotypes (dbGaP) under the project ID (pending - submitted to local IRB and NCI, April 2020). Results from variant calling were then filtered using the “vcffilter” function found in the C++ library tool vcflib (https://github.com/vcflib/vcflib#vcflib). For MuTect2 results, variants were filtered based on the MuTect2-assigned filter flag, quality score, and read depth (FILTER = PASS, QUAL > 20 and DP > 20). For freebayes results, tumor and germline variants were separated into individual vcf files using “vcfsamplediff” function in vcflib. For freebayes somatic results, variants were filtered based on the quality score, read depth, and somatic score fields (QUAL > 20, DP > 20 and SSC > 50). For freebayes germline results, variants were filtered based on quality score and read depth (QUAL > 20 and DP > 20). The filtered variants were annotated using ANNOVAR release version “2018Apr16” [54]. The following databases were used for annotation: RefSeq, dbsnp build 150, ExAC v0.3, and gnomAD exomes v2.1.1. Using ANNOVAR’s “annotate_variantion.pl” script, the annotated variants were further filtered based on the gnomAD annotation using a score threshold of 0.05, leaving only variants with a population frequency <5% remaining.

### 4.4. Pathway and Double Hit Analysis

Using vcftools, the remaining variants were restricted to either known oncosignaling genes for pathway analysis [28] or known oncogenes [55] and tumor suppressor genes [56] for the double hit analysis. Mutations in these gene sets were assessed for driver status using the Cancer Genome Interpreter (CGI) [57].

### 4.5. Copy Number Alteration Analysis

Somatic copy number alterations (CNAs) were identified using Sequenza v2.1.2 [22] for R (v3.5.1). CNAs were matched to chromosomal bands and gene locations using a custom R script using the packages GenomicRanges v1.36.0 [58], annotatr v1.10.0 [59], org.Hs.eg.db v3.8.2 [60], and biomaRt v2.40.0 [61]. Amplified and deleted regions were checked for overlap with identified driver mutations to add to the double-hit analysis. Heat maps showing the frequency of amplifications/deletions in 2.5 Mbp windows along the chromosomes were generated using the R package chromoMap v0.2 [62]. Sequenza results files were then used as input to GISTIC 2.0 for comparison with gene-level results from The Cancer Genome Atlas (TCGA, National Institutes of Health, Bethesda, MD, USA). Parameters used for GISTIC 2.0 were based on those reported for the TCGA project except where they had to be modified to reflect the greater noisiness of our WES-derived segment calls as compared to the array-based data used by the TCGA. Gene identifiers from NCBI were assigned by GISTIC using the included hg19 reference file. Counts of protein-coding genes were then compiled for the entire gene set, as well as for lists of oncogenes [55] and tumor suppressor genes [56].

## 5. Conclusions

WES of tumor-normal allows for better CNV analysis in addition to identifying germline pathogenic mutations. In addition, WES of rare tumors provides information that is outside the current search space of gene panels but may ultimately provide clinically useful data to be used now or in the future. Although there is currently a relative shortage of tumor-specific FDA approved therapies and clinical trials for rare tumor patients, as novel targeted therapies become more readily available, specification of somatic vs. germline mutations and CNA in newly linked cancer genes will be critical in determining the best possible treatment options. This is especially important given the possibility of positive treatment outcomes for rare tumors with targeted and immune checkpoint therapies. Taken together, our study highlights the urgent need for exploration of tumorigenesis pathways in rare tumors and the subsequent need for development of specific clinical trials to determine optimal treatment strategies. As shown in our study, rare tumor patients may disproportionately benefit from both tumor-germline and WES approaches to potentially allow for better treatment options via future tumor genome-guided discoveries and therapies.

## Figures and Tables

**Table 1:** Filtered germline and somatic driver mutations.

Germline TSG Drivers								
Case	Ref	Alt	Gene	ExonicFunc.refGene	avsnp150	gnomAD Freq.	Polyphen2	PhyloP
4	G	A	MAD1L1	nonsyn. SNV	rs121908982	0.0036	Prob. Damaging	5.785
7	C	G	IGF2R	nonsyn. SNV	rs8191844	0.0102	Prob. Damaging	4.24
7	C	-	BRCA2	frameshift deletion	rs80359718	N/A	N/A	N/A
13	G	A	NUP98	nonsyn. SNV	rs61751338	0.0014	Prob. Damaging	6.327
13	T	C	TET2	nonsyn. SNV	rs144386291	0.0055	Prob. Damaging	6.829
2	C	T	EPHA2	nonsyn. SNV	rs34192549	0.0123	Benign	0.073
12	TGGTGAAGAACATTCAGGCAA	-	BARD1	inframe deletion	rs28997575	0.024	N/A	N/A
5	C	T	EPHA2	nonsyn. SNV	rs34192549	0.0123	Benign	0.073
5	G	A	IGF2R	nonsyn. SNV	rs8191753	0.0023	Prob. Damaging	3.63
1	C	T	EXT2	nonsyn. SNV	rs138495222	0.0006	Prob. Damaging	7.813
14	G	A	AXIN2	nonsyn. SNV	rs138287857	0.0012	Poss. Damaging	7.551
15	G	C	LZTS1	nonsyn. SNV	rs148775156	0.001	Prob. Damaging	2.853
15	G	A	PTCH1	nonsyn. SNV	rs138911275	0.0007	Poss. Damaging	7.645
11	G	A	ARID2	nonsyn. SNV	rs200040222	0.0001	Prob. Damaging	9.317
10	C	A	DLC1	nonsyn. SNV	.	N/A	Prob. Damaging	2.753
3	TGGTGAAGAACATTCAGGCAA	-	BARD1	inframe deletion	rs28997575	0.024	N/A	N/A
3	C	T	CTNND1	nonsyn. SNV	rs199813020	0.0003	Prob. Damaging	2.101
8	G	A	STARD13	nonsyn. SNV	rs144801804	0.0008	Prob. Damaging	9.889
8	C	T	SDHA	stopgain	rs781764920	6.37E-05	N/A	2.131
8	-	AGG	FBXW7	inframe insertion	rs541979458	0.0014	N/A	N/A
8	C	T	ATM	nonsyn. SNV	rs28904919	0.0014	Poss. Damaging	3.491
16	A	C	NOTCH2	nonsyn. SNV	rs147223770	0.0032	Poss. Damaging	8.947
16	C	T	ATM	nonsyn. SNV	rs56009889	0.0004	Prob. Damaging	3.766
17	G	A	CHEK2	nonsyn. SNV	rs730881690	N/A	Prob. Damaging	8.668
18	C	T	EPHA2	nonsyn. SNV	rs34192549	0.0123	Benign	0.073
18	-	GCGGGT	CEBPA	inframe insertion	rs762459325	0.0416	N/A	N/A
20	G	A	BRCA2	nonsyn. SNV	rs41293503	N/A	Prob. Damaging	3.408
20	C	G	KMT2C	nonsyn. SNV	rs138119145	0.0065	Prob. Damaging	6.729
20	C	T	ATR	nonsyn. SNV	.	N/A	Poss. Damaging	6.179
21	T	C	DICER1	nonsyn. SNV	rs747510783	N/A	Prob. Damaging	7.502
21	T	C	ATM	nonsyn. SNV	.	N/A	Poss. Damaging	6.589
21	A	G	MSH2	nonsyn. SNV	rs773177076	N/A	Prob. Damaging	9.147
25	T	C	TET2	nonsyn. SNV	rs144386291	0.0055	Prob. Damaging	6.829
25	T	C	NUP98	nonsyn. SNV	rs201011075	0.0005	Prob. Damaging	4.516
22	A	G	LZTS1	nonsyn. SNV	rs149140637	0.0031	Prob. Damaging	7.441
22	C	T	LATS1	nonsyn. SNV	rs148506316	0.0002	Benign	5.885
23	G	T	PPP2R5C	nonsyn. SNV	rs147942579	0.0003	Prob. Damaging	9.014
24	A	G	SDHB	nonsyn. SNV	rs771004483	N/A	Poss. Damaging	8.563
24	C	T	EPHA2	nonsyn. SNV	rs139787163	0.0003	Prob. Damaging	1.668
24	C	T	GPC3	nonsyn. SNV	rs11539789	0.003	Benign	2.917
24	A	G	SDHD	nonsyn. SNV	rs11214077	0.0048	Prob. Damaging	2.521
26	C	T	SMARCA4	nonsyn. SNV	rs763471007	N/A	Prob. Damaging	7.813
26	G	A	TSC1	nonsyn. SNV	rs878853968	N/A	Prob. Damaging	8.039
27	C	T	EPHA2	nonsyn. SNV	rs34192549	0.0123	Benign	0.073
27	-	GCGGGT	CEBPA	inframe insertion	rs762459325	0.0416	N/A	N/A
19	T	A	MCC	nonsyn. SNV	rs17313892	0.0076	Prob. Damaging	4.64
19	C	T	EPHA2	nonsyn. SNV	rs34192549	0.0123	Benign	0.073
13	G	A	TXNIP	nonsyn. SNV	rs781868836	N/A	N/A	4.673
**Germline Oncogene Drivers**								
Case	Ref	Alt	Gene	ExonicFunc.refGene	avsnp150	gnomAD Freq.	Polyphen2	PhyloP
13	G	A	NUP98	nonsyn. SNV	rs61751338	0.0014	Prob. Damaging	6.327
13	C	T	CSF1R	nonsyn. SNV	rs138432536	0.003	Poss. Damaging	3.03
2	C	T	EPHA2	nonsyn. SNV	rs34192549	0.0123	Benign	0.073
12	TGGTGAAGAACATTCAGGCAA	-	BARD1	inframe deletion	rs28997575	0.024	N/A	N/A
5	C	T	EPHA2	nonsyn. SNV	rs34192549	0.0123	Benign	0.073
1	G	C	MUC4	nonsyn. SNV	rs369326402	0.0001	Poss. Damaging	−1.2
14	C	T	MET	nonsyn. SNV	rs34589476	0.0025	Benign	3.567
15	G	A	PTCH1	nonsyn. SNV	rs138911275	0.0007	Poss. Damaging	7.645
9	G	A	NSD2	nonsyn. SNV	rs758343111	N/A	Poss. Damaging	3.202
10	T	-	CHD1L	stopgain	rs781989601	N/A	N/A	N/A
3	TGGTGAAGAACATTCAGGCAA	-	BARD1	inframe deletion	rs28997575	0.024	N/A	N/A
3	GGAGCTCCATCC	-	TRIO	inframe deletion	rs140308852	0.0076	N/A	N/A
18	T	G	PDGFRA	nonsyn. SNV	rs148654387	0.0004	Poss. Damaging	6.111
18	C	T	EPHA2	nonsyn. SNV	rs34192549	0.0123	Benign	0.073
18	C	T	MYC	nonsyn. SNV	rs200431478	0.0002	Prob. Damaging	4.735
25	G	C	MUC4	nonsyn. SNV	rs369326402	0.0001	Poss. Damaging	−1.2
25	T	C	NUP98	nonsyn. SNV	rs201011075	0.0005	Prob. Damaging	4.516
24	G	A	FIP1L1	nonsyn. SNV	rs777738679	N/A	Prob. Damaging	7.569
24	C	T	EPHA2	nonsyn. SNV	rs139787163	0.0003	Prob. Damaging	1.668
26	G	C	MUC4	nonsyn. SNV	rs369326402	0.0001	Poss. Damaging	−1.2
27	C	T	EPHA2	nonsyn. SNV	rs34192549	0.0123	Benign	0.073
19	T	A	MCC	nonsyn. SNV	rs17313892	0.0076	Prob. Damaging	4.64
19	G	C	MUC4	nonsyn. SNV	rs369326402	0.0001	Poss. Damaging	-1.2
19	C	T	EPHA2	nonsyn. SNV	rs34192549	0.0123	Benign	0.073
**Somatic TSG Drivers**								
Case	Ref	Alt	Gene	ExonicFunc.refGene	avsnp150	gnomAD Freq.	Polyphen2	PhyloP
4	G	A	TP53	nonsyn. SNV	rs876659802	N/A	Prob. damaging	10.003
13	C	-	ARID1A	frameshift deletion	.	N/A	N/A	N/A
13	G	A	CREBBP	stopgain	.	N/A	N/A	6.795
2	G	C	FOXL2	nonsyn. SNV	rs1057519865	N/A	Prob. damaging	2.22
12	C	T	KMT2C	nonsyn. SNV	rs145833795	9.55E-05	Prob. damaging	4.525
1	A	-	RB1	frameshift deletion	.	N/A	N/A	N/A
14	G	A	TP53	nonsyn. SNV	rs587780070	N/A	Prob. damaging	10.003
14	A	T	TP53	nonsyn. SNV	rs1057519982	N/A	Prob. damaging	9.325
15	G	A	CDKN2A	stopgain	rs121913388	N/A	Benign	1.26
15	G	C	IDH1	nonsyn. SNV	rs121913499	N/A	Poss. damaging	7.103
9	G	A	IDH1	nonsyn. SNV	rs121913499	N/A	Benign	7.103
10	C	A	TP53	stopgain	rs201744589	N/A	N/A	−0.746
19	-	T	SETD2	frameshift insertion	.	N/A	N/A	N/A
19	AAG	-	CTCF	inframe deletion	.	N/A	N/A	N/A
7	TCATCTCA	-	TNFAIP3	frameshift deletion	.	N/A	N/A	N/A
**Somatic Oncogene Drivers**								
Case	Ref	Alt	Gene	ExonicFunc.refGene	avsnp150	gnomAD Freq.	Polyphen2	PhyloP
7	GGAG	-	CHD1L	frameshift deletion	rs782573713	N/A	N/A	N/A
2	G	C	MUC4	nonsyn. SNV	rs369326402	0.0001	Poss. damaging	−1.2
15	G	C	IDH1	nonsyn. SNV	rs121913499	N/A	Poss. damaging	7.103
9	G	A	IDH1	nonsyn. SNV	rs121913499	N/A	Benign	7.103
3	G	T	HRAS	nonsyn. SNV	rs28933406	N/A	Benign	9.821
3	G	A	PIK3CA	nonsyn. SNV	rs121913273	N/A	Prob. damaging	9.602
16	C	T	EGFR	nonsyn. SNV	rs149840192	N/A	Prob. damaging	7.882
20	G	A	PIK3CA	nonsyn. SNV	rs121913273	N/A	Prob. damaging	9.602
19	AGTGGA	-	KIT	inframe deletion	rs869025568	N/A	N/A	N/A

**Table 2:** ACMG classified pathogenic/likely pathogenic variants detected.

Case ID	Cancer Type	Gene	Mutation Type	Coding Change	Protein Change	gnomAD Freq	Polyphen2	PhyloP	ACMG Classification
7	Gray Zone Lymphoma	BRCA2	Frameshift	NM_000059.3: c.8575delC	NP_000050.2: p.Gln2859Lysfs	N/A	N/A	N/A	Pathogenic
8	Breast Cancer, Spindle Cell	SDHA	Nonsense	NM_004168.3: c.223C>T	NP_004159.2: p.Arg75Ter	6.47E-05	N/A	2.131	Pathogenic
12	GIST	SDHC	Frameshift	NM_003001.3: c.6delT	NP_002992.1: p.Ala3Argfs	N/A	N/A	N/A	Pathogenic
14	Glioblastoma	RUNX1	Missense	NM_001754.4: c.451A>T	NP_001745.2: p.Met151Leu	N/A	N/A	N/A	Likely Pathogenic
16	Glioma (Anaplastic Astrocytoma)	FANCC	Splice Region (Exon Skipping)	NM_000136.2: c.456+4A>T	N/A	0.0001	N/A	N/A	Pathogenic
18	Aveolar Soft Part Sarcoma	MUTYH	Missense	NM_001128425.1: c.1187G>A	NP_001121897.1: p.Gly396Asp	0.0032	Prob. damaging	4.511	Pathogenic

**Table 3:** Inherited pathogenic variants in common and rare cancers.

Cancer Case Set	Total Cases	Pathogenic/Likely Pathogenic Germline Variant	No Pathogenic Germline Variant
Rare Cancer (UAGC)	27	6 (22.2%)	21 (77.8%)
Common Cancer (Total)	3451	274 (7.9%)	3177 (92.1%)
Fisher Exact Test *p*-value, UAGC vs. Total Common: 0.01800			
Common Cancers	Total Cases	Pathogenic/Likely Pathogenic Germline Variant	No Pathogenic Germline Variant
CRC [21]	141	27	114
Breast [21]	85	16	69
Prostate [21]	26	3	23
NSCLC [21]	33	4	29
SCLC [21]	11	0	11
COAD (colon) [14]	419	25	394
READ (rectal) [14]	145	6	139
BRCA (breast) [14]	1076	106	970
PRAD (prostate) [14]	498	27	471
LUAD (lung adeno) [14]	518	33	485
LUSC (lung squamous) [14]	499	27	472
Common - Total	3451	274	3177

**Table 4:** Pathogenic/Likely Pathogenic Germline Variants detected across studies.

Rare Cancers	Total Cases	Pathogenic/Likely Pathogenic Germline Variant		No Pathogenic Germline Variant	
UAGC Rare Cancer	27	6 (22.2%)		21 (77.8%)	
Huang et al. [14] Rare Cancer	1955	163 (8.3%)		1792 (91.7%)	
Bertelsen et al. [21] Rare Cancer	122	22 (18.0%)		100 (82.0%)	
[14] + [21] Rare Cancer	2077	185 (8.9%)		1892 (91.1%)	
Fisher Exact Test *p*-value, UAGC vs. [14] + [21]: 0.03035					
**Huang et al. [14] Rare Cancers:**	**Total Cases**	**Pathogenic/Likely Pathogenic Germline Variant**		**No Pathogenic Germline Variant**	
Adrenocortical Carcinoma		92	4		88
Cholangiocarcinoma		45	1		44
Glioblastoma Multiforme		393	23		370
Brain Lower Grade Glioma		515	31		484
Mesothelioma		82	7		75
Pheochromocytoma and Paraganglioma		179	41		138
Sarcoma		255	32		223
Thymoma		123	6		117
Uterine Carcinosarcoma		57	2		55
Uveal Melanoma		80	4		76
Testicular Germ Cell Tumors		134	12		122
Sum		1955	163		1792
**Bertelsen et al. [21] Rare Cancers:**		**Total Cases**	**Pathogenic/Likely Pathogenic Germline Variant**		**No Pathogenic Germline Variant**
Bile Duct Cancer		47	8		39
Sarcoma		14	3		11
Neuroendocrine Cancer		13	1		12
Malignant Mesothelioma		12	7		5
Adrenocortical Cancer		8	0		8
Thymoma		8	1		7
Adenoid Cystic Carcinoma		5	1		4
Myoepithelial Carcinoma		4	0		4
Glioblastoma		4	0		4
Anogenital Cancer		3	0		3
Vulvovaginal Cancer		2	1		1
Germ Cell Cancer		2	0		2
Sum		122	22		100

## Supplementary Materials

The following are available online at https://www.mdpi.com/2072-6694/12/6/1618/s1, Figure S1: CNA gene counts from TCGA rare and common cancer cohorts; Figure S2: CNA gene counts from TCGA rare and common cancer cohorts, broken down by cancer type; Table S1: 1670 were found in oncogenes and 1673 were in tumor suppressor genes, generating an average of 124 germline variants per case; Table S2: The number of somatic SNVs and small indels detected in all cases totaled 523, with 306 in oncogenes and 217 in tumor suppressor genes; Table S3: Classification of oncogenic genes (OG) and tumor suppressor genes (TSG) was defined by the Oncogene; Table S4: Total number of amplified oncogenes and deletions of tumor suppressor genes in the cohort rare cancer cases; Table S5: Drivers genes with additional copy number variation—Double Hits; Table S6: GO Enrichment analysis.

## Abbreviations

ACC	Adrenocortical carcinoma
CHOL	Cholangiocarcinoma
GBM	Glioblastoma multiforme
LGG	Brain Lower Grade Glioma
MESO	Mesothelioma
PCPG	Pheochromocytoma and Paraganglioma
SARC	Sarcoma
TGCT	Testicular Germ Cell Tumors
THYM	Thymoma
UCS	Uterine carcinoma
UVM	Uveal melanoma
BRCA	Breast invasive carcinoma
COAD	Colon adenocarcinoma
LUAD	Lung adenocarcinoma
LUSC	Lung squamous cell carcinoma
PRAD	Prostate adenocarcinoma
READ	Rectum adenocarcinoma
ACMG	American College of Medical Genetics
