# Supplementary material for: Rare Tumor-Normal Matched Whole Exome Sequencing Identifies Novel Genomic Pathogenic Germline and Somatic Aberrations
Source: Cancers (Basel). 2020 Jun 18;12(6):1618. doi: 10.3390/cancers12061618 (PMC7352311; doi:10.3390/cancers12061618)

Supplementary Figures and Tables

Sprissler RS et al.

Figure S1: CNA gene counts from TCGA rare and common cancer cohorts. Gene-level results produced by GISTIC were downloaded from the cBioPortal. Only high-level amplifications (red) and putative homozygous deletions (blue) were included. **A)**. All protein-coding genes. **B)**. Oncogenes. **C)**. Tumor suppressor genes.

Figure S1A

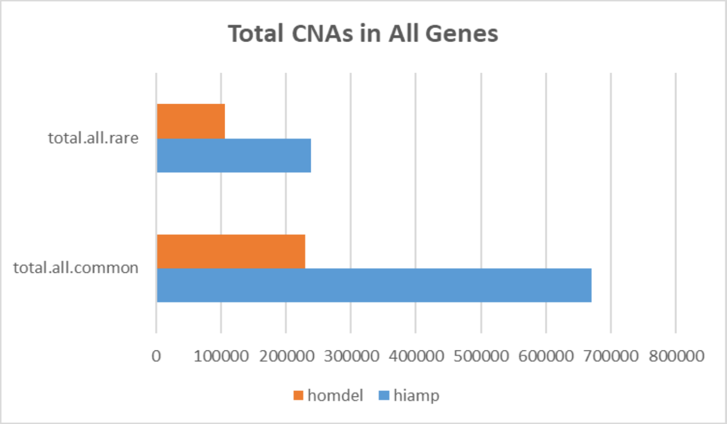

Figure S1B

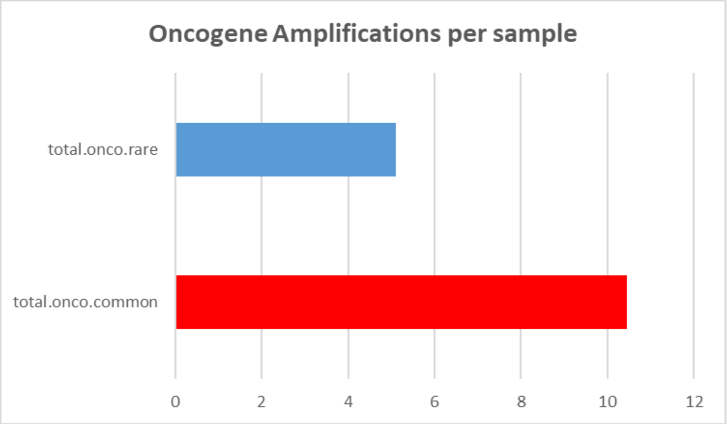

Figure S1C

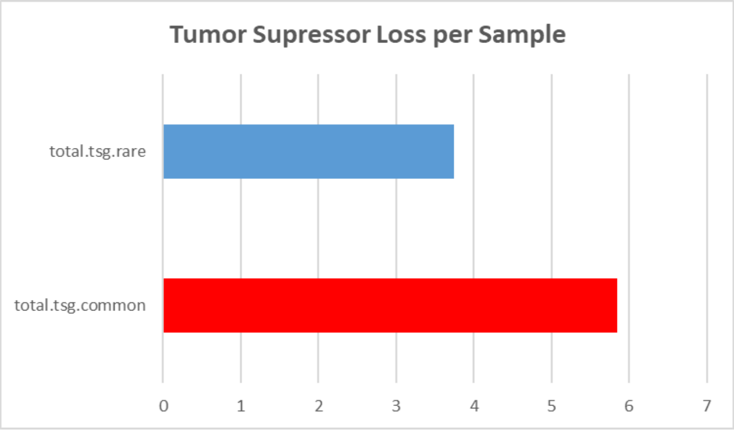

Figure S2. CNA gene counts from TCGA rare and common cancer cohorts, broken down by cancer type. **A)**. All protein-coding genes. **B)**. Oncogenes. **C)**. Tumor suppressor genes.

Figure S2A

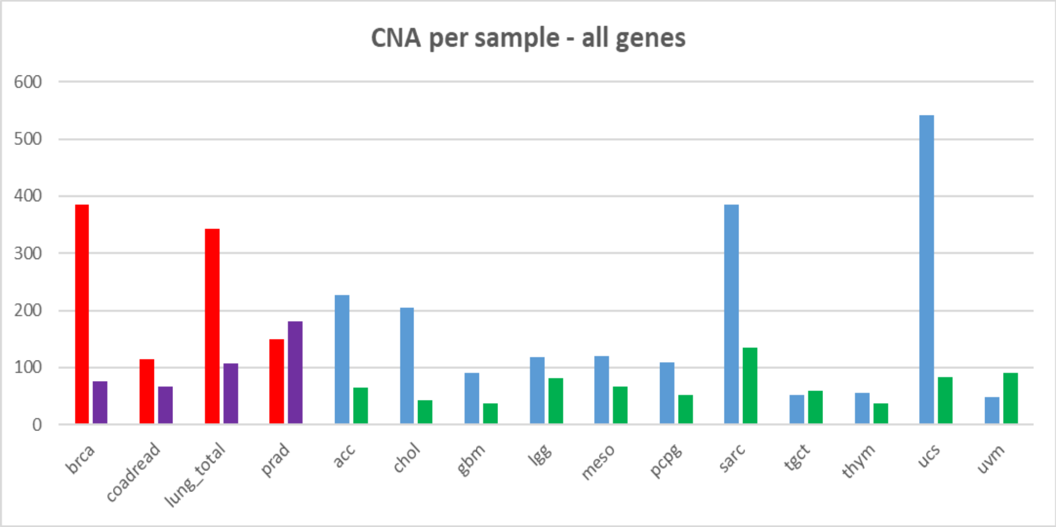

Figure S2B

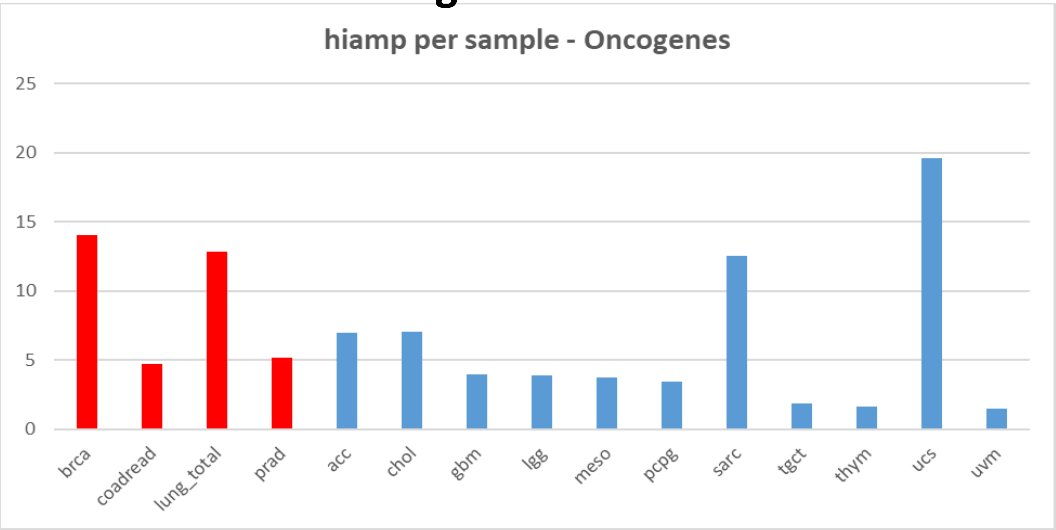

Figure S2C

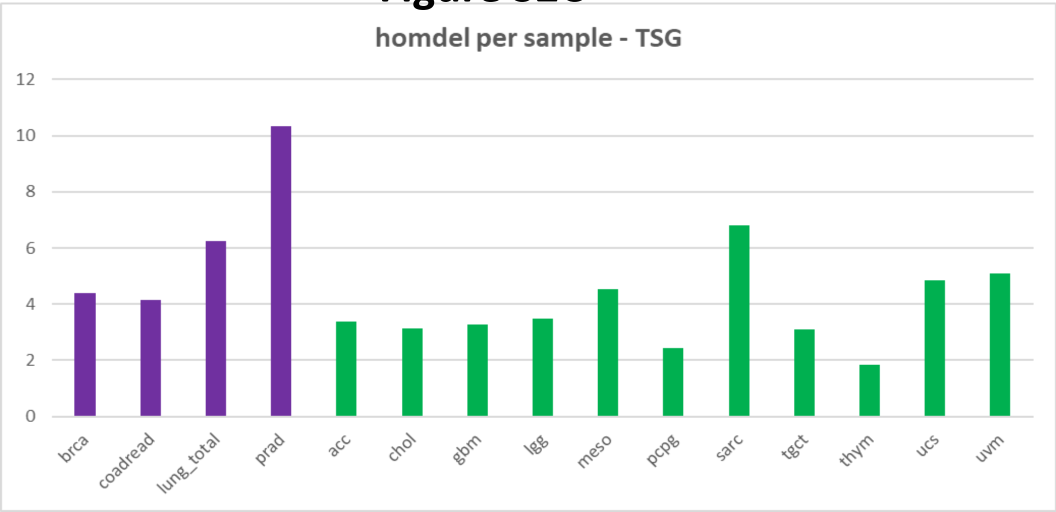

Supplement: Supplementary file 1 [file cancers-12-01618-s001.zip › cancers-817200-Supplementary Materials3/cancers-817200-supplementary figure.pdf]
